# P-343. The Feasibility of Safely and Efficiently Administering Antivirals to Nursing Home Residents During COVID-19 Outbreaks is Impaired by Polypharmacy and Comorbidities

**DOI:** 10.1093/ofid/ofae631.545

**Published:** 2025-01-29

**Authors:** Amelia Milner, Brigid Wilson, Oteshia Hicks, Taissa A Bej, Corinne Kowal, Jennifer Pruskowski, Federico Perez, Robin Jump

**Affiliations:** University of Pittsburgh, Fairview Park, Ohio; VA Northeast Ohio Healthcare System, Cleveland, Ohio; VA Northeast Ohio Healthcare System, Cleveland, Ohio; Louis Stokes Cleveland VA Medical Center, Cleveland, Ohio; Louis Stokes Cleveland VA Medical Center, Cleveland, Ohio; VA Pittsburgh Healthcare System, Pittsburgh, Pennsylvania; Case Western Reserve University, Cleveland, OH; VA Northeast Ohio Healthcare System, Cleveland, Ohio

## Abstract

**Background:**

Due to congregate living, advanced age, and frailty, nursing home (NH) residents are at high risk for infections caused by respiratory viruses. Protocols for the efficient and safe administration of antivirals for the treatment and prophylaxis of influenza among NH residents are well-established. Similar protocols for COVID-19 are foreseeable. Use of nirmatrelvir/ritonavir, associated with improved outcomes in high-risk patients with COVID-19, requires careful examination of concomitant medications, renal function, and blood pressure. Here, we explore the potential to administer nirmatrelvir/ritonavir safely and efficiently to NH residents in response to COVID-19 outbreaks.

Maximum Severity of Drug Interaction with nirmatrelvir/ritonavir

**Figure:**
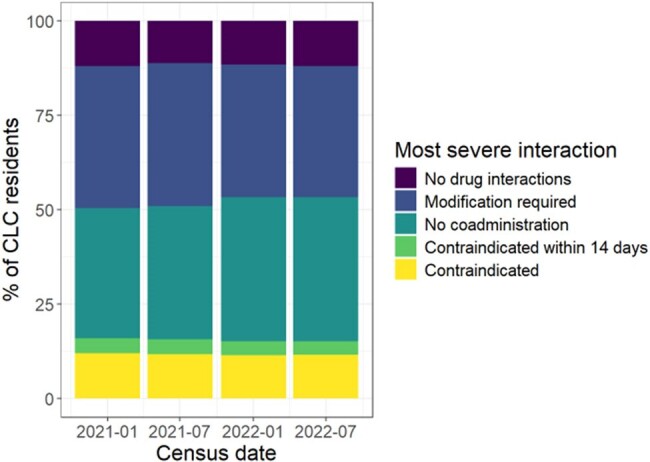

The percentage of NH patients taking at least one medication within 14 days of the census date is categorized by the maximum severity of drug interaction with nirmatrelvir/ritonavir.

**Methods:**

We studied residents of NHs within the Veterans Health System at four census dates in 2021-2022. We assessed residents’ medications over the prior 14 days, the most recent estimated glomerular filtration rate (eGFR), and vitals for each date. We calculated the proportion of residents with and without potential interactions between their medication regimen and nirmatrelvir/ritonavir. We also determined the proportion of residents with conditions that require dosing adjustments or exacerbate drug interactions such as renal impairment and hypotension.

eGFR Values among Nursing Home Patients

**Figure:**
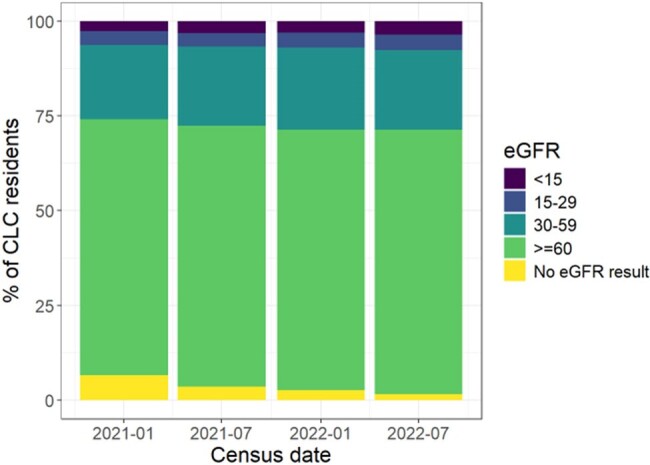

Renal function is represented as the percentage of NH patients at each census date categorized by eGFR values.

**Results:**

The median number of distinct drugs per NH resident in the prior 14 days was 13 (IQR: 10-17). Among the >99% of residents with at least one medication administered 14 days before each census date, 88% received at least one agent with an interaction or contraindication with nirmatrelvir/ritonavir (Figure). Among the 50 most commonly prescribed medications, 7 required significant dose modifications. At all 4 census dates, ≥25% of NH residents received atorvastatin, tamsulosin, or amlodipine. Similarly, ≥25% of residents had eGFR < 60mL/minute and 9-10% had blood pressure < 90/60 mmHg.

Hypotension Percentages among Nursing Home Patients

**Figure:**
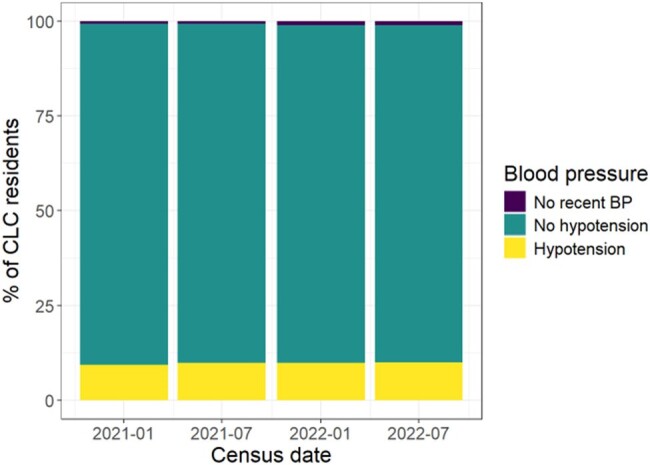

Each bar represents the percentage of NH patients with hypotension at that census date. Blood pressure analysis was used to determine hypotension with a threshold value of <90/60 mmHg.

**Conclusion:**

The prevalence of polypharmacy and renal impairment among NH residents challenges the widespread administration of nirmatrelvir/ritonavir as a safe and efficient measure during COVID-19 outbreaks. Clinical decision support tools embedded within electronic health records may help mitigate these risks, as will the availability of effective antiviral agents with fewer drug-drug interactions.

**Disclosures:**

**Federico Perez, MD, MS**, Merck: Grant/Research Support **Robin Jump, MD, PhD**, Abacus: Grant/Research Support|Merck: Grant/Research Support|Pfizer: Advisor/Consultant|Pfizer: Grant/Research Support

